# Perceptions of interprofessional collaborative practice in South Africa: A systematic review

**DOI:** 10.4102/hsag.v29i0.2413

**Published:** 2024-02-29

**Authors:** Nadia Mohamed, Craig W. Peck, Janine Senekal

**Affiliations:** 1 Department of Paediatric Dentistry, Faculty of Dentistry, University of the Western Cape, Cape Town, South Africa; 2 Research Development and Postgraduate Support, University of the Western Cape, Cape Town, South Africa

**Keywords:** barriers, enablers, healthcare, healthcare professionals, interprofessional education, interprofessional collaborative practice, South Africa, systematic review

## Abstract

**Background:**

Interprofessional education (IPE) and interprofessional collaborative practice (IPCP) were developed to address the health needs of communities through collaborative practice across healthcare disciplines. The impact of IPE on IPCP and clinical service delivery in South Africa is not evident, possibly because of the lack of IPCP experiences among healthcare professionals.

**Aim:**

International literature reports facilitators and barriers of IPCP implementation, but there was a need to filter the evidence to identify literature from the South African context regarding the perceptions of healthcare workers’ perceived barriers and facilitators of IPCP.

**Setting:**

South African literature.

**Methods:**

A systematic review was conducted to synthesise evidence from articles published between January 2017 and December 2021. Only qualitative studies targeting health professionals in South Africa who had been exposed to IPCP were included. Consistent with Preferred Reporting Items for Systematic reviews and Meta-Analysis, a multi-database search yielded 424 articles, which were screened for relevance and appraised for quality using the Critical Appraisal Skills Programme (CASP) tool. A thematic synthesis of the findings was conducted by applying ethical principles.

**Results:**

Synthesis of barriers and enablers for IPCP implementation in the South African context included key aspects of healthcare systems, management and team leadership.

**Conclusion:**

The integration of IPCP into clinical practice in South Africa is still limited as healthcare professionals operate in silos.

**Contribution:**

Recommendations of this study include greater integration of services combined with competent management and visionary leadership, together with the incorporation of IPE into undergraduate professional training programmes.

## Introduction

Interprofessional collaborative practice (IPCP) refers to the healthcare team working together to provide comprehensive patient care (World Health Organization [WHO] 2010) by combining a range of competencies and skills and facilitating the optimal use of resources (Samuelson et al. 2012). It is the intentional and goal-directed sharing of information and resources, which has the ability to change the attitudes and behaviours of healthcare professionals towards each other and their respective functions. This culminates in better patient care by regarding the patient as the ultimate stakeholder (Schmitt et al. 2011). The IPCP team may involve professionals from different disciplines, the patient, their family members and various other stakeholders. Interprofessional collaborative practice implementation is dependent on a multitude of interconnected aspects and dimensions such as communication, teamwork and the acknowledgement of individual roles and responsibilities (Verhaegh et al. 2017).

The concepts of interprofessional education (IPE) and IPCP were introduced more than 40 years ago as a means to address the complex health needs of communities through collaboration between professionals across all areas of health. In 2010, the WHO (2010) called for the optimisation of healthcare equity through improved IPE and collaborative practice. In order for IPCP to be realised, the education and learning thereof must precede it, which would then create opportunities to promote collaboration. A transformative scale-up within health professions education (HPE) was therefore initiated internationally with the inclusion of IPE in curricula (Van Heerden 2013; WHO 2011). Health professions education should focus on delivering healthcare professionals who are competent collaborators of education (via IPE) and patient-centred practitioners (via IPCP) within their respective communities (Matthews & Naidu 2019; Oandasan & Reeves 2005). Even if students and health professionals are trained to work collaboratively, this might not necessarily translate into action to improve health outcomes, reduce the cost of care and improve the patient experience (Bierwas et al. 2017; Ellapen et al. 2018). It is therefore important to identify the perceptions of healthcare professionals with regard to barriers and facilitators of IPCP as these might provide reasons for ineffective or insufficient IPCP implementation.

The incorporation of IPCP into clinical practice is limited, both locally and internationally (Bierwas et al. 2017; Kock, Mlezana & Frantz 2021). As a result, the impact of IPE on IPCP and clinical service delivery is not evident where the overall health of the population is concerned. This could be because of the complexities within the healthcare system as well as the experiences of health professionals with IPCP, which could be hampering its uptake (Brandt et al. 2014). Although IPCP and its implementation are reported to be more impactful in developed countries, healthcare targets have still not been met (Herath et al. 2017). The lack of impact of IPE and IPCP on healthcare targets is even greater in developing countries, which raises questions as to how effectively IPE has been translated to IPCP in reality (Herath et al. 2017). The barriers to IPCP implementation could therefore be higher within the South African context. The current South African National Policy has made provision for the core principles and outcomes of IPCP within the 2030 National Development Plan, in support of addressing healthcare inequality by promoting equitable healthcare outcomes for all (National Planning Commission 2011). It is therefore relevant to identify perceptions of healthcare professionals in the South African context specifically, in order to delineate barriers and facilitators to IPCP implementation.

There is evidence of systematic reviews that have been conducted on IPE (Hammick et al. 2007; Herath et al. 2017; Mann, Gordon & MacLeod 2009) and the implementation of IPCP. However, the focus of these has generally been on patient outcomes (Reeves et al. 2013). Even though the international literature has reported on facilitators and barriers of IPCP, the perceptions of healthcare workers towards IPCP within the South African context have not yet been the subject of a systematic review. Contextual factors needed to be taken into consideration when examining the implementation of IPCP. It is important to consider healthcare workers’ perceptions towards interprofessional communication and collaboration, as this directly impacts the delivery of effective IPCP (Verhaegh et al. 2017).

Through understanding what the perceptions of healthcare workers towards IPCP in the South African context are, key areas to improve patient care and treatment outcomes, reduce medical costs and reduce treatment errors are identified.

## Research question

What are the perceptions of healthcare workers towards IPCP and what are the perceived barriers and facilitators of IPCP in South Africa?

## Aim

While there is evidence of the value of IPCP in improving patient outcomes, there was a need to filter literature as it relates to healthcare professionals’ perceptions of IPCP and within the South African context as opposed to the global context. For this reason, a systematic review was conducted with the aim of collating and consolidating empirical evidence regarding healthcare professionals’ perceptions of IPCP in South Africa.

## Rationale for the systematic review

Systematic reviews are considered to be secondary research, which are protocol-driven and quality focussed. They are regarded as the highest form of summarising evidence – including within healthcare (Gopalakrishnan & Ganeshkumar 2013). Systematic reviews identify all studies that meet the inclusion criteria with a clearly stated set of objectives, adopting an explicit and reproducible methodology that makes them rigorous by design (eds. Higgins & Green 2011).

## Research methods and design

A systematic review was conducted to consolidate and synthesise the evidence from the literature regarding healthcare workers’ perceptions of IPCP in their local South African work contexts. To ensure unambiguousness and transparency, the Preferred Reporting Items for Systematic reviews and Meta-Analysis (PRISMA) guidelines were adopted for this study (Page et al. 2021). As such, the review followed the process of screening, eligibility, appraisal and synthesis. The study obtained ethical clearance from the Humanities and Social Science Research Ethics Committee of the University of the Western Cape.

### Inclusion and exclusion criteria

Inclusion criteria were defined in advance, to systematically determine which articles were to be included. The criteria for this study were as follows:

- Text selection: Only peer-reviewed, full-text, English articles were included.
- Time period: Articles published between January 2017 and December 2021 were included.
- Population or target group: Healthcare professionals involved in patient care who had been exposed to IPCP in the South African context were included.
- Type of studies: Only primary qualitative articles were included, given that this systematic review is focused on the perceptions of IPCP.
- Outcome: There must have been an evaluation or indication of the perceptions of these healthcare workers towards IPCP, with regard to barriers and facilitators of IPCP.

Articles published in a foreign language, or those translated into English from another language, were excluded in order to ensure that there was no ambiguity within the content of the articles (i.e. misinformation because of possible differences regarding interpretation). Grey literature (dissertations and theses, conference papers, discussion forums) and predatory literature were excluded from the study, with the intention of using only high-quality, peer-reviewed articles from reputable journals. Journals without formal editorial or review boards and those where information regarding the peer-review process, fees and expertise of the editorial board members was lacking were excluded. Articles where the full text could not be accessed were excluded as the full text was needed for evaluation, appraisal and data extraction.

### Search strategy

The study was conducted between March and July 2022. Keywords were identified based on the population, issue, context and outcome (PICO). The keywords were then used to develop Boolean phrases which are combinations of keywords that are more effective in literature searches than single keywords or search terms (Muhammad 2017). A composite search of multiple databases (including Springer Link, EBSCOhost, Science Direct, Wiley Online Library, Elsevier Science Direct) was conducted through uKwazi, which is available through the University of the Western Cape library. All available databases were searched for articles simultaneously, the results of which automatically excluded duplicate articles while simultaneously removing duplicates. The Boolean phrases were entered into uKwazi for a composite search (Table 1), and filters for the relevant time period (January 2017 to December 2021), resource type (article), access type (available online) and peer review status (peer-reviewed) in alignment with the inclusion criteria were applied.

**TABLE 1:** Search strategy.

Adapted PICO	Search terms
Population	Health professionals OR Healthcare workers
Issue	IPCP OR Interprofessional Collaborative Practice
Context	South Africa
Outcome	Barriers OR Enablers OR Facilitators OR Perceptions OR Experiences

PICO, population, issue, context and outcome.

### Screening and selection

The search results were exported from uKwazi and imported into Rayyan, which is a free online systematic review software that streamlines the review process, allowing multiple reviewers to work simultaneously and automatically collating reasons for inclusion and exclusion (Ouzzani et al. 2016).

Titles and abstracts were screened for relevance and eligibility against the inclusion and exclusion criteria. Screening was a dual review process, done by three investigators independently, followed by a process of collaboration to reach consensus and ensure the rigour of article inclusion. Using three independent reviewers in this study improved the methodological validity before the inclusion of the articles, thereby meeting the methodological requirements of a systematic review (Heyvaert, Hannes & Onghena 2017). Disagreement was dealt with through discussion with a fourth reviewer. The remaining full-text articles were sourced and screened for eligibility and relevance based on the inclusion and exclusion criteria. The reasons for exclusion were recorded, and disagreement between the three reviewers was resolved through discussion, with motivation.

The remaining articles were critically appraised for quality and rigour using the CASP tool (Critical Appraisal Skills Programme [CASP] 2018) for qualitative studies, as only qualitative studies were included. Laher and Hassem (2020) provided a guide as to how to interpret the findings, and this was used to set the threshold levels for inclusion of articles. For qualitative studies, the upper threshold of quality is 10. Scores between 7 and 10 were included, while studies scoring between 4 and 6 were deliberated. Studies that scored below 4 were excluded. Each article was appraised by at least two reviewers, and the results of the critical appraisal process were recorded (Table 2).

**TABLE 2:** Critical Appraisal Skills Programme quality appraisal score of included articles.

Author	Publication date	Average appraisal score	Decision
Kock et al.	2021	8	Include
Maddocks et al.	2017	7	Include
Ntinga & Van Aswegen	2020	8.5	Include
Nyoni et al.	2021	8.5	Include
Waggie & Arends	2020	8.5	Include

### Data extraction and analysis

The relevant descriptive and analytic data were extracted from the studies selected for inclusion using a self-developed data extraction table. The descriptive data focussed on the context of the study, details of the participants or sample, and methods that were employed. The analytic data included those aspects that have direct bearing on the research question (e.g. perceptions of IPCP as well as barriers and/or facilitators to implementing IPCP). Thematic synthesis was used to collate the information obtained from reviewing all the articles (Thomas et al. 2017). Coding words were generated, and emerging themes across the various studies were identified deductively to facilitate comparison (Thomas et al. 2017). The descriptive themes that emerged were then synthesised to answer the review question (Thomas et al. 2017).

### Ethical considerations

The study obtained ethical clearance from the Humanities and Social Science Research Ethics Committee of the University of the Western Cape (Ethical clearance number: HS22/6/25).

## Results

### Data search findings

**FIGURE 1:**
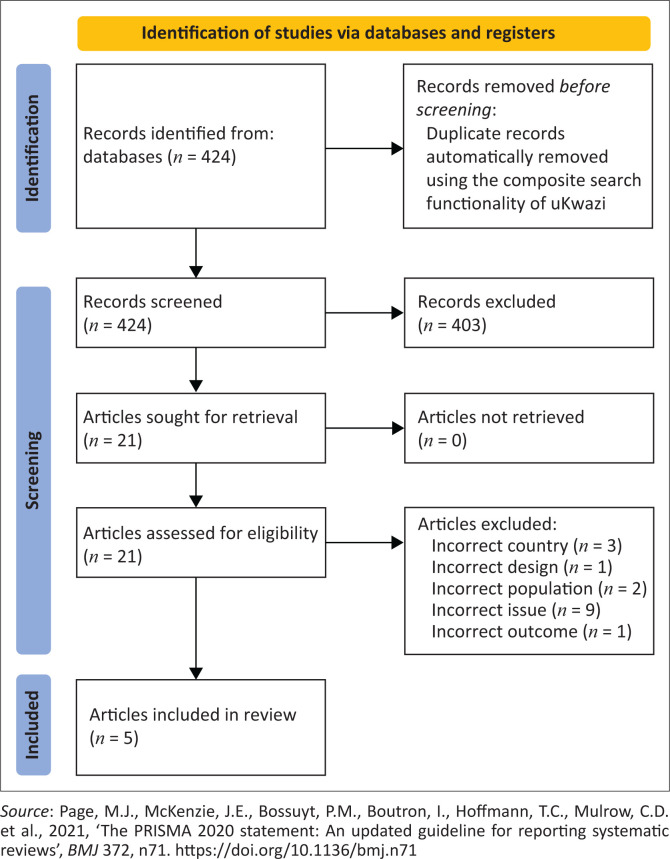
Preferred Reporting Items for Systematic reviews and Meta-Analysis 2020 flow diagram for new systematic reviews that included searches of databases and registers only.

A total of 424 articles were identified, after applying filters, in the composite search which automatically excluded any duplicates (Figure 1). In the title and abstract phase, 403 articles were excluded from the study according to the predefined inclusion and exclusion criteria. The remaining 21 articles were downloaded in full text and were independently reviewed for eligibility and relevance by two reviewers. A further 16 articles which did not meet the predetermined inclusion criteria were excluded. The five remaining articles underwent quality appraisal.

### Results of the quality appraisal

The five selected articles were critically appraised for quality and rigour using the CASP tool (CASP 2018) for qualitative studies. All five articles scored between 7 and 8.5 (Table 2), which are considered strong scores that may be included (Laher & Hassem 2020).

### Descriptive data results

#### Geographic location and setting

All the studies were conducted in South Africa, as per the inclusion criteria (Table 3). The articles covered two public hospitals (Nyoni, Grobler & Botma 2021; Waggie & Arends 2020), a community healthcare centre (Kock et al. 2021), a semi-rural hospital (Maddocks et al. 2018) and intensive care units (ICUs) in the public and private sectors (Ntinga & Van Aswegen 2020).

**TABLE 3:** Descriptive data summary.

Authors	Geographic location	Setting	Study design	Sampling	Sample size	Data collection methods	Health professionals involved
Kock et al. 2021	Nyanga, South Africa	Primary Community Health Centre	Qualitative, exploratory, descriptive, case study	Voluntary participation	33 participants	Four semi-structured focus groups	Physicians, nurses, radiographers, pharmacists, physiotherapists, OTs, dieticians, social workers, healthcare promoters, administrative staff (from different departments at the same facility)
Maddocks et al. 2018	KwaZulu-Natal, South Africa	Semi-rural Hospital	Qualitative, descriptive	Purposive sampling Voluntary	Eight participants	Semi-structured interviews	Doctors, physiotherapists
Ntinga & Van Aswegen 2020	Johannesburg, South Africa	Urban ICU Four private and four public sector hospitals	Qualitative (mostly) and quantitative (using descriptive statistics)	Convenience sampling Voluntary	39 participants	Semi-structured focus groups	Junior and senior physiotherapists
Nyoni et al. 2021	Free State, South Africa	Central hospital Low or limited resource setting	Qualitative Observation	Purposive selection	Four wards	Unstructured observation Reflections Author-generated transcripts	Doctors, nurses, physiotherapists, nutritionists, speech therapists, caregivers of patients
Waggie & Arends 2020	Western Cape, South Africa	Tertiary public hospital	Qualitative, descriptive, exploratory	Purposive selection using pre-criteria	14 participants	Focus groups	Medical doctors, physiotherapists, OTs, social workers, dieticians, speech and language therapists, nurses

OTs, Occupational therapists.

#### Methodological information

All the studies were qualitative in nature with only one study employing a small quantitative component (Ntinga & Van Aswegen 2020). Three articles used focus group discussions (Kock et al. 2021; Ntinga & Van Aswegen 2020; Waggie & Arends 2020), while Nyoni et al. (2021) employed unstructured observational research and Maddocks et al. (2018) used privately conducted interviews with participants. These types of data collection methods allowed for the in-depth exploration of issues under investigation, making it possible for points to be clarified with follow-up questions, thereby adding richness to the information obtained. Either purposive (Maddocks et al. 2018; Nyoni et al. 2021; Waggie & Arends 2020) or convenience (Ntinga & Van Aswegen 2020; Kock et al. 2021) sampling techniques were used where researchers relied on voluntary participation of the study subjects.

#### Health professionals

All professionals included in the studies were part of interprofessional teams. Three studies included professionals from a range of different professional groups (Kock et al. 2021; Nyoni et al. 2021; Waggie & Arends 2020). However, it must be kept in mind that one study was an observational study where the authors observed the interaction between professionals from two or more different professions (Nyoni et al. 2021). One study only involved doctors and physiotherapists (Maddocks et al. 2018) and another study only examined the perceptions of junior and senior physiotherapists working as part of an interprofessional team in intensive care unit (ICU) facilities at private and public hospitals (Ntinga & Van Aswegen 2020). In four of the studies, professionals reported on their lived experiences as they were frontline workers who were part of an interprofessional team and were directly involved with patient care. The study by Nyoni et al. (2021) was the only observational study where the health professionals were not directly involved in the generation of data.

### Analytic data results

#### Healthcare workers’ perceptions of practising and implementing interprofessional collaborative practice

Healthcare professionals’ perceptions of IPCP were summarised under two themes related to the barriers and enablers of IPCP. Barriers to IPCP implementation included healthcare system, team structure, team processes, team relationships, and management and leadership. Facilitators to IPCP implementation included team processes, team relationships, and management and leadership.

#### Perceived barriers to practising and implementing interprofessional collaborative practice

**Healthcare system:** The healthcare system plays a vital role in facilitating IPCP into clinical practice, and several barriers in the healthcare system which hamper IPCP were identified in the five studies. The high burden of disease and large patient numbers means that many facilities have to cope with fewer staff (Kock et al. 2021; Nyoni et al. 2021; Waggie & Arends 2020), leaving less time for IPCP (Kock et al. 2021; Maddocks et al. 2018). Staff shortages, increased workload and associated stresses leave staff feeling overwhelmed as primary healthcare services are target driven (Waggie & Arends 2020). The shortage of staff, high patient loads and time constraints were issues that were common to all five studies. This was noted as the main reason why the inclusion of the patient in the care plan (as is typical in IPCP) is often neglected (Waggie & Arends 2020; Nyoni et al. 2021). Low-resource settings usually do not have access to health professionals from all the various disciplines (Maddocks et al. 2018; Nyoni et al. 2021) as staff sometimes must rotate between different facilities. Scheduling timetables between the different professions and coordination of leave are not easy, leaving fewer staff to participate in IPCP at a particular time (Kock et al. 2021; Maddocks et al. 2018). The distance between departments, lack of a common space to have discussions and infrastructural barriers like broken telephones present further barriers to collaboration (Kock et al. 2021; Nyoni et al. 2021). Various aspects within the healthcare system and facilities presented practical barriers to IPCP implementation.

**Team structure:** Hierarchical culture was the overriding factor referred to in each of the five articles as a barrier to IPCP where, according to Nyoni et al. (2021:8), ‘professional identity mirrors professional hierarchy’. Three studies identified that doctors tended to dominate patient care and took the lead in decision-making, while other team members felt undervalued (Maddocks et al. 2018; Nyoni et al. 2021; Waggie & Arends 2020). Waggie and Arends (2020) and Maddocks et al. (2018) also reported that doctors dominated patient care and took the lead in decision-making, while other team members were undervalued. Physiotherapists felt that their tasks often had to take a backseat as the more dominant professions (like medical doctors) tended to interrupt their work (Ntinga & Van Aswegen 2020). Hierarchical conflicts were also more commonly reported by junior physiotherapists, in relation to their senior counterparts (Ntinga & Van Aswegen 2020). The discrepancies in salaries, different levels of education and amount of experience vary considerably between members of interprofessional teams, reinforcing the hierarchical culture (Nyoni et al. 2021). Team structure is also determined by the number and variety of health professionals available to work in an interprofessional context (Kock et al. 2021; Nyoni et al. 2021). Patients were not considered as part of the team in any of the studies. This is contrary to IPCP principles, which place the patient at the centre of care and include them in decisions involving their own health (WHO 2010). As such, the nature of the team structure, including the traditional hierarchical culture, was a barrier to IPCP implementation.

**Team processes:** Three of the five studies highlighted the lack of knowledge of the roles and scope of practice of the various professionals in the interprofessional team and the lack of communication between the team members as being barriers that affected team processes (Waggie & Arends 2020; Maddocks et al. 2018; Nyoni et al. 2021). Observations of an interprofessional team revealed that very little discussion took place between team members, with documented patient case notes being the point of reference (Nyoni et al. 2021). The language of communication within the interprofessional team was also listed as a barrier, as this hampered communication, resulting in possible misinterpretation of information pertaining to patient management (Nyoni et al. 2021; Waggie & Arends 2020). Professionals did not engage in collaborative goal-setting (Waggie & Arends 2020) and still tended to operate in silos (Nyoni et al. 2021; Waggie & Arends 2020). Various barriers to communication thus disrupted the implementation of IPCP team processes.

**Team relationships:** Four of the articles stressed the importance of attitudes as barriers or enablers to the collaborative process (Kock et al. 2021; Ntinga & Van Aswegen 2020; Nyoni et al. 2021; Waggie & Arends 2020). Frequent rotation of staff does little to cement good relationships between team members as they do not get to know each other well (Ntinga & Van Aswegen 2020). Overlapping the scope of practice or disregard for professional boundaries resulted in a power struggle that can exacerbate professional jealousy (Nyoni et al. 2021; Waggie & Arends 2020). Nyoni et al. (2021) observed clear dominant and subservient behaviour patterns between doctors and nurses, highlighting the unequal contribution to the decision-making process. In addition, having different perceptions of teamwork contributed to a tense working environment (Ntinga & Van Aswegen 2020). A negative attitude towards IPCP can hamper team relationships if team members do not recognise the benefit of IPCP (Waggie & Arends 2020). A lack of discussion and collaboration between professionals and a lack of clarity regarding the roles of the various professionals within the team can result in misunderstandings regarding the treatment that each of the disciplines is expected to carry out. This results in poor patient outcomes and, in turn, reinforces professionals’ negative perceptions of IPCP (Kock et al. 2021). As such, the nature of team relationships was a barrier to the implementation of IPCP.

**Management and leadership:** Poor leadership was also identified as a barrier to the implementation of IPCP in two of the studies (Ntinga & Van Aswegen 2020; Waggie & Arends 2020). Swift conflict resolution and provision of continued professional development (CPD) opportunities were seen as essential duties of an effective team manager (Ntinga & Van Aswegen 2020). A lack of leadership training contributed to poor management (Waggie & Arends 2020).

#### Perceived enablers of practising and implementing interprofessional collaborative practice

The enablers of practising and implementing IPCP were implied through the suggestions and recommendations that participants in the articles under review highlighted. In most instances, these were not current practices, but rather suggestions or recommendations made by healthcare practitioners of what would improve their ability to operate collaboratively.

**Team processes:** Two studies identified specific aspects of team processes that may be greatly improved by encouraging input from all members of the interprofessional team (Maddocks et al. 2018; Ntinga & Van Aswegen 2020). Improving communication and knowledge of the scope of practice of individuals within the team (Maddocks et al. 2018) may facilitate the sharing of knowledge (Ntinga & Van Aswegen 2020). Use of goal-orientated tools (like WhatsApp groups for sharing pictures and videos) may improve collaboration (Waggie & Arends 2020) as patient information and treatment goals can be communicated effectively and discussed collectively by all members of the team (Ntinga & Van Aswegen 2020). Having all team members present at one time and in one place would also facilitate IPCP (Ntinga & Van Aswegen 2020). The above may improve collaborative team processes and thus enable IPCP implementation.

**Team relationships:** Two studies identified that improving team relationships, through mutual respect, trust, friendliness and a positive disposition towards team members, would go a long way towards opening lines of communication (Maddocks et al. 2018; Ntinga & Van Aswegen 2020). Encouraging professionals to focus on a common goal may help to limit the pervasive hierarchical culture and encourage a teamwork approach to patient care (Ntinga & Van Aswegen 2020). Improved communication and the development of good interpersonal relationships would result in better teamwork (Waggie & Arends 2020). By recognising the importance of mutually beneficial relationships between team members with a variety of knowledge, skills and experience, staff could develop more positive attitudes towards IPCP (Waggie & Arends 2020). These practices of improving team relationships may support the implementation of IPCP.

**Management and leadership:** Managers can influence the work environment through their actions and the structures that they put in place (Williams, Van Rooyen & Ricks 2018). Good management and leadership are therefore essential enablers of IPCP. This includes the ability to manage conflict and create opportunities for growth and development (Ntinga & Van Aswegen 2020).

## Discussion

The review synthesised evidence in the South African context of healthcare professionals’ perceptions of barriers and enablers to IPCP implementation. Challenges were encountered at the healthcare systems level, the interprofessional team level and the level of the individual practitioner, all of which are needed to enable effective collaboration.

### Healthcare system

All five articles mentioned the healthcare system as a barrier to IPCP in the respective contexts, mentioning that staffing issues, high patient turnover and high workloads limit the opportunity for IPCP in the workplace (Kock et al. 2021; Maddocks et al. 2018; Ntinga & Van Aswegen 2020; Nyoni et al. 2021; Waggie & Arends 2020). The South African health system is heavily burdened, presenting with inadequate human and financial resources (Maddocks et al. 2018; Nyoni et al. 2021), poor infrastructure and inadequate space for optimal IPCP. Particularly in the public hospital and community healthcare clinic settings (Kock et al. 2021; Maddocks et al. 2018), working environments are not conducive to or designed for IPCP because of a lack of privacy (Nyoni et al. 2021). In resource-constrained settings in particular, large patient numbers and staff shortages mean that the remaining staff at the facilities have to take on tasks that fall outside their scope of practice, compounding the stress staff experience and increasing their workload (Williams et al. 2018). Inadequate facilities and failing communication technologies add to staff frustration. The latter hampers communication considerably, especially when professionals are not housed in the same facility (Williams et al. 2018). Allowing professionals from different disciplines to share the same workspace enables collaboration (Kates et al. 2011) and ensures that representatives from the different professions are present at any given time, thereby facilitating the IPCP process (Rawlinson et al. 2021; Supper et al. 2014). These international issues related to IPCP are well aligned with those found in the studies under review. As such, these are not unique to the South African context, but rather common issues related to IPCP implementation. The role of the patient in the collaborative process was, however, not evident in the South African settings that were analysed.

### Team processes and relationships

Where the healthcare professional was concerned, the overriding issues involved team processes and team relationships. Issues of hierarchy and poor attitudes of team members are common in the literature dealing with interprofessional collaboration (Supper et al. 2014). Hierarchy reinforces traditional stereotyping and limits healthy collaborative engagement through dominance and power struggles (Minamizono et al. 2013; Van Winkle et al. 2013). Administrative and educational structures as well as professional cultures encourage this hierarchical culture where certain professions enjoy superiority in terms of authority, status and income. Hierarchical dominance gives rise to power struggles that are sustained by the nature of the referral process (Kock et al. 2021) and leads to poor communication (Kock et al. 2021; Ntinga & Van Aswegen 2020; Waggie & Arends 2020). Poor communication and a lack of adequate discussion affected the development of meaningful and sustainable working relationships between team members (Kock et al. 2021; Maddocks et al. 2018; Ntinga & Van Aswegen 2020; Nyoni et al. 2021; Waggie & Arends 2020). There is general agreement in the literature that improving communication would go a long way towards enhancing IPCP (Rawlinson et al. 2021; Supper et al. 2014). Language barriers should also be addressed, particularly in the South African context where there are 11 official languages. Despite communication being identified as a key barrier to IPCP in this study, it was similarly cited by all five articles under review as a vital enabler for IPCP going forward. A common language, free of professional jargon, should be used in an IPCP setting to enhance communication, especially regarding treatment protocols and processes that need to be followed. Clarity on the scope of practice of the various professions is needed to provide clear guidelines and eliminate confusion in the context of collaboration (Ambrose-Miller & Ashcroft 2016; Yusra, Ardi Findyartinib & Soemantrib 2019). A lack of awareness of colleagues’ scope of practice is a common issue that has been highlighted in the international literature (Brown et al. 2014; Hall 2005). Tools can be used to improve communication regarding operational processes, and regular meetings can help to foster an atmosphere of mutual trust and respect among team members (Rawlinson et al. 2021). By developing a shared vision, each team member can take ownership of their role in the IPCP process (Rawlinson et al. 2021) and, in so doing, help to temper issues of hierarchy (Ambrose-Miller & Ashcroft 2016). Focussing on the patient’s needs could also diminish hierarchical conflicts (Price et al. 2006 cited by Supper et al. 2014).

### Practitioner understanding of interprofessional collaborative practice

Interprofessional collaborative practice is best explained as a move away from a multidisciplinary approach (Benagiano & Brosens 2014) and towards integrated holistic patient care (Ford & Gray 2021). From the articles reviewed, there seemed to be a misconception regarding what IPCP entailed. Kock et al. (2021) highlighted the fact that professionals thought they were engaging in IPCP when in fact it was more likely that they were practising in a multidisciplinary fashion that involves independent functioning of each discipline (Benagiano & Brosens 2014). Referrals between health professionals were considered interprofessional practice (IPP) when in fact, each discipline was still functioning in isolation. Maddocks et al. (2018) concurred with this assessment. There was also a lack of integration of interprofessional practice into clinical care (Kock et al. 2021). Additionally, professionals did not recognise the benefits of IPCP and did not understand the negative effect this had on patient outcomes. The importance of management and leadership in interprofessional settings was thus highlighted. Williams et al. (2018) noted that managers did not necessarily have the motivation to bring about change. Team leaders have to recognise the value of IPCP and be willing to promote it by empowering staff and creating environments that would enable the integration of interprofessional collaboration into clinical practice.

## Recommendations

After analysing these perceptions, it became clear that the majority were not unique to the South African context, but rather showed similarities to international research. Several recommendations were highlighted in the articles under review to facilitate the uptake of IPCP among professionals, especially in the South African context. These are discussed below.

### Healthcare system

A primary healthcare system that functions effectively will reduce pressure at a tertiary level (Waggie & Arends 2020). Waggie and Arends (2020) highlighted the importance of increased human resources within the health system, which would facilitate IPCP without further negative impact on workload and patient care. Increasing the number of staff and providing incentives to retain staff would help to reduce patient loads, thereby reducing the stress on existing staff (Waggie & Arends 2020; Williams et al. 2018). Each health facility should ideally have a wide range of staff from different disciplines (Williams et al. 2018). If this is not possible, solutions should be found to ensure adequate staff coverage or treatment times should be structured to ensure that various team members are available when needed (Ntinga & Van Aswegen 2020). Nyoni et al. (2021:10) suggested that ‘trans-professional models of care’ be introduced which would expand the scope of practice of some disciplines, thereby stretching resources, which would be particularly applicable to resource-limited settings. However, it might also add to the stress of these individuals and would require upskilling of these staff to enable them to confidently perform tasks outside their scope of practice (Williams et al. 2018). Workspaces should be created which would make collaboration and discussion easier (Kock et al. 2021; Waggie & Arends 2020). Co-location or housing different professions in the same building would help to facilitate this.

### Team structure

An investment should be made into introducing interventions to address the hierarchical culture in the healthcare sector, as this is a major barrier to IPCP (Waggie & Arends 2020). Such interventions should encourage shared decision-making (Waggie & Arends 2020) and could involve case studies and simulations so that professionals can practise their IPCP skills (Nyoni et al. 2021). Such interventions would also assist with role clarification and establishing of protocols for collaborative goal setting (Waggie & Arends 2020) and may also address the overlap or duplication of tasks (Nyoni et al. 2021).

### Team processes

Introduction of common assessment tools, checklists and structured protocols may reduce confusion, improve communication and encourage collaboration (Maddocks et al. 2018; Ntinga & Van Aswegen 2020). Information about roles and scope of practice, care pathways and processes should be readily accessible to all members of the interprofessional team and visible in all the wards (Maddocks et al. 2018; Ntinga & Van Aswegen 2020; Waggie & Arends 2020). This would enable better patient care through improved IPCP efforts. Additionally, improved staff orientation (Ntinga & Van Aswegen 2020) and continued IPE efforts that promote transformational learning within the IP team (Maddocks et al. 2018; Nyoni et al. 2021) would also enable IPCP.

### Team relationships

An inclusive environment that fosters respect should be created (Waggie & Arends 2020). Shared decision-making processes should empower team members to put forward their ideas confidently. For example, team-building activities and initiatives would help to improve collaboration between team members from different backgrounds (Nyoni et al. 2021).

### Management and leadership

Managers of facilities should take it upon themselves to raise awareness among their staff regarding teamwork and collaboration (Williams et al. 2018). By including ‘working in an interprofessional team’ in the job description, staff may be more aware of what is expected of them (Waggie & Arends 2020). There should be a move away from referrals (which are instructional by nature) and a move towards a more collaborative approach to patient care (Kock et al. 2021). Managers should also provide staff with opportunities (such as continued professional development activities) to upskill themselves and acquire competencies to improve collaboration (Kock et al. 2021). It is important that these activities are scheduled strategically to avoid staff shortages at any given time (Nyoni et al. 2021).

## Strengths and limitations

A systematic review of this nature focussing on the perceptions of IPCP of health workers in South Africa has not been previously conducted and thus adds to the body of knowledge by providing a synthesis of contextual barriers and facilitators to IPCP, as well as recommendations for improving IPCP implementation. By nature, all reviews are limited to their specific focus and inclusion and exclusion criteria. This review focused specifically on healthcare professionals’ perceptions of barriers and enablers of IPCP and not on IPCP itself. As such, the results do not consider the participants’ existing feelings about IPCP itself or their level of experience or understanding of IPCP, which may influence their perceptions of barriers and enablers to its implementation.

## Conclusion

The importance of interprofessional collaboration is increasingly being highlighted as patient needs are becoming more complex. Its implementation can however be challenging. Based on the definition of IPCP which involves health professionals from different professional backgrounds working together with the patient to provide comprehensive patient care, it would appear as though its integration into clinical practice settings in South Africa is still limited by many contextual barriers such as resources and infrastructure in the health system itself. These barriers are complex to address, given the political and organisational obstacles that need to be overcome to address them.

Professions still seem to be operating in silos as opposed to combining their unique range of competencies to facilitate holistic patient management. Barriers and facilitators to the integration of IPCP into clinical practice have been highlighted, including resource and infrastructural challenges and difficulties at a team level where individual team members are involved. Where certain healthcare systems’ challenges might be difficult to change in the short term, barriers on an individual practitioner level might be easier to address. The concept of IPCP needs to be entrenched in the healthcare system by making professionals aware of its value and the part that each professional can play in improving collaborative practices, especially on an individual practitioner level where attitudes of mutual respect and cooperation can be fostered, and the role of the ego takes a backseat in these professional relationships. Healthcare reform that is IPCP focussed will need institutional and governmental intervention of the many barriers to IPCP that are reported both nationally and internationally. Greater integration of services, competent management and leaders with vision are needed to make inroads into the integration of IPCP within healthcare settings.

It is vital to introduce IPCP as early as possible before power differentials between individuals grow too large. Therefore, it is important to focus on training undergraduate students with an eye on producing health professionals who are aware of IPCP and are able to incorporate it into practice. Providing context-specific IPE training for undergraduate students from different disciplines and IPE during in-service training would plant the seed for IPCP and encourage students to engage and collaborate with their peers. Behaviour and attitudes of students can be shaped to develop professionals who are familiar with IPCP and appreciate its value. For qualified professionals, CPD activities to introduce the concepts of IPE and provision of opportunities for interprofessional practice (that do not take up a lot of time) can reinforce the IPCP culture. Team leaders and managers should also be actively involved in establishing IPCP and improving interprofessional team dynamics so that professionals develop a positive attitude towards IPCP. This would ultimately result in an improvement in the quality of care that patients receive.
